# Itraconazole exerts its anti-melanoma effect by suppressing Hedgehog, Wnt, and PI3K/mTOR signaling pathways

**DOI:** 10.18632/oncotarget.15324

**Published:** 2017-02-14

**Authors:** Guanzhao Liang, Musang Liu, Qiong Wang, Yongnian Shen, Huan Mei, Dongmei Li, Weida Liu

**Affiliations:** ^1^ Department of Mycology, Institute of Dermatology, Chinese Academy of Medical Sciences and Peking Union Medical College, Nanjing, China; ^2^ Georgetown University Medical Center, Washington, DC, USA

**Keywords:** itraconazole, melanoma, hedgehog, Wnt, mTOR

## Abstract

Malignant melanoma is the deadliest form of all skin cancers. Itraconazole, a commonly used systemic antifungal drug, has been tested for its anti-tumor effects on basal cell carcinoma, prostate cancer, and non-small cell lung cancer. Whether itraconazole has any specific anti-tumor effect on melanoma remains unknown. However, the goal of this study is to investigate the effect of itraconazole on melanoma and to reveal some details of its underlying mechanism. In the *in vivo* xenograft mouse model, we find that itraconazole can inhibit melanoma growth and extend the survival of melanoma xenograft mice, compared to non-itraconazole-treated mice. Also, itraconazole can significantly inhibit cell proliferation, as demonstrated by Ki-67 staining in itraconazole-treated tumor tissues. In *in vitro*, we show that itraconazole inhibits the proliferation and colony formation of both SK-MEL-28 and A375 human melanoma cells. Moreover, we demonstrate that itraconazole significantly down-regulates Gli-1, Gli-2, Wnt3A, β-catenin and cyclin D1, while it up-regulates Gli-3 and Axin-1, indicating potent inhibitory effects of itraconazole on Hedgehog (Hh) and Wnt signaling pathways. Furthermore, itraconazole significantly suppresses the PI3K/mTOR signaling pathway – indicated by the down-regulated phosphorylation of p70S6K, 4E-BP1 and AKT – but has no effect on the phosphorylation of MEK or ERK. Our data suggest that itraconazole inhibits melanoma growth through an interacting regulatory network that includes Hh, Wnt, and PI3K/mTOR signaling pathways. These results suggest that this agent has several potent anti-melanoma features and may be useful in the synergesis of other anti-cancer drugs via blockage of the Hh, Wnt and PI3K/mTOR signaling pathways.

## INTRODUCTION

Malignant melanoma is one of the most aggressive cancers, accounting for roughly 4% of human skin cancers but producing approximately 80% of deaths due to cutaneous neoplasms [1]. The outcome of patients with metastatic melanoma remains very poor, with a 5-year survival rate of only 5%–15%, a rate which has not seen any significant improvement despite intensive therapeutic efforts over many decades [2].

Activated mutations in the oncogenes B-RAF (the RAS-regulated kinase) and N-RAS have been detected in 50-70% and 15%-30% of melanoma patients, respectively [3]. Some genetic alterations on signaling molecules such as CDKN2A, PDK1, PTEN, and AKT have also been associated with the pathogenesis of melanoma [4]. It is well known that the inappropriate reactivation of key developmental signaling pathways in cancer is a common signature, of which Hh and Wnt pathways are prime examples [5]. Other recent studies have confirmed that Hh and Wnt signal transduction pathways are relevant to the formation and progression of melanoma [6, 7]. Consequently, efforts to focus on development of inhibitors targeting these pathways are critically important for cancer treatment, and in particular melanoma.

Itraconazole, an FDA-approved agent belonging to the family of antifungal drugs known as azoles, has been used clinically as such for more than 30 years. In recent years, based on antiangiogenic and antitumor activities that were inferred from several *in vitro* and *in vivo* models [8–10], this compound has been repurposed in a number of phase 2 clinical trials for cancer treatment. To date, investigations of the effectiveness of itraconazole on basal cell carcinoma, prostate cancer, and non-small cell lung cancer have shown that itraconazole can increase the progression-free survival of these cancer patients [11–13]. In addition, retrospective studies in patients with recurrent triple-negative breast cancer and ovarian cancer showed significant increases of overall survival rate when itraconazole was involved [14, 15]. These results suggest that itraconazole has a great potential for becoming a new anti-tumor drug.

Despite these promising clinical results, the molecular mechanism by which itraconazole inhibits tumors remains largely unknown. One possibility is that itraconazole can inhibit angiogenesis and endothelial cell proliferation by targeting VDAC1 to modulate the AMPK/mTOR signaling axis in endothelial cells [16]. However, angiogenesis is already known not to be a dominant factor for the progression of melanoma [17]. Recently, itraconazole has also been proposed as an antagonist of hedgehog (Hh) signaling pathway that targets protein Smoothened (SMO), which regresses basal cell carcinoma (BCC) and medulloblastoma. The SMO receptor, which is composed of an extracellular cysteine-rich domain (CRD) and an ECD linker domain, shows a high sequence similarity to the frizzled (FZD) receptors. The latter contain an extracellular domain (ECD) as well, which mediates the Wnt signaling pathway [18, 19]. Indeed, SMO and FZD receptors have been classifies in the same class [20, 21]. As the structures between SMO and FZD are similar, a possibility arises that itraconazole may target both Hh and Wnt signaling pathways in melanoma.

To better understand the molecular mechanisms of itraconazole on tumors, the effects of itraconazole on melanoma tumor growth in mouse and *in vitro* cell proliferation are assessed and the influences of itraconazole on cell developmental signaling pathways are investigated. We find that itraconazole effectively inhibits melanoma by suppressing Hh, Wnt and PI3K-mTOR signaling pathways.

## RESULTS

### Itraconazole inhibits proliferation and colony formation of melanoma cells

**Figure 1:**
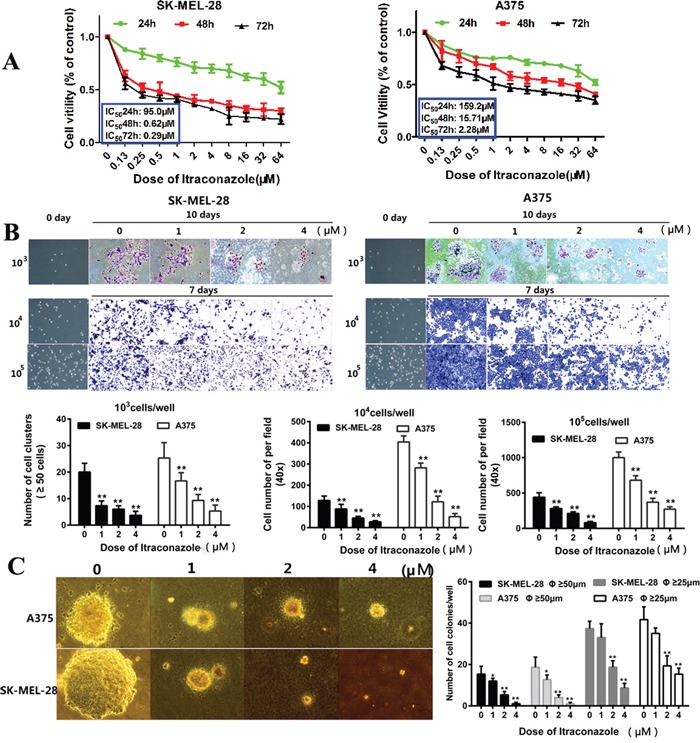
Inhibitory effect of itraconazole on A375 and SK-MEL-28 cells proliferation **A**. CCK-8 assay showed suppression of cell viability in A375 and SK-MEL-28 cells. A375 and SK-MEL-28 melanoma cells were cultured in various concentrations of itraconazole for 24, 48, 72h. Results are expressed as percentage of inhibition of growth compared with control, *P<0.05 and **P<0.01. **B**. Representative photographs of cell numbers and quantitative analysis of cell proliferation. A375 and SK-MEL-28 cells were seeded at different density (10^3^, 10^4^ and 10^5^cell/well), treated with itraconazole (0, 1, 2, and 4 μM) for 48 h and cultured for additional 5 days or 7 days. At the end of incubation, cells were stained with crystal violet solution. The cell number was counted and analyzed. The experiments were repeated three times with consistent results. **P<0.01 versus control group. **C**. Representative photographs of cell colonies and quantitative analysis. A375 and SK-MEL-28 cells were treated with itraconazole (0, 1, 2, and 4 μM) for 14 days in soft agar. At the end of incubation, pictures were taken (200×). Colonies with diameter ≥50μm or ≥25μm were counted and statistically analyzed. *P<0.05 and **P<0.01 versus control group.

To understand the effects of itraconazole on the proliferation of A375 and SK-MEL-28 melanoma cells, we use three methods to measure cell growth and viability. First, tumor cells were incubated with different concentrations of itraconazole ranging from 0.13 to 64 μM, and CCK-8 assay was used to detect cell viability at the indicated time points (24-, 48- and 72-hr post treatment as shown in Figure 1A). Itraconazole-untreated cells at each time point were used as controls. We find that itraconazole can effectively suppress the proliferation of A375 and SK-MEL-28 cells in both a time- and a dose-dependent manner, with approximate IC50 (95% CI) values of 159.2 μM (24h, 58.23–435.2 μM), 15.71 μM (48h, 10.06–24.54 nM), and 2.28 μM (72h, 1.56–3.34μM) in A375 cells and 95.0 μM (24h, 53.82–167 μM), 0.62 μM (48h, 0.2928-1.329 μM) and 0.29 μM (72h, 0.1332-0.6455 μM) in SK-MEL-28 cells, respectively (Figure 1A). The overall inhibitory effects of itraconazole seem more evident in SK-MEL-28 cells than in A375 cells with an inhibitory plateau reached at 48 hr.

Second, the proliferation of tumor cells was observed under microscopy by a cell counting assay. In the absence of itraconazole, A375 and SK-MEL-28 cells rapidly proliferate and begin to form cell clusters at 48 hr. However, both types of tumor cells display reduced proliferation after treatment with 1, 2 or 4 μM itraconazole for 7-10 days. As shown in Figure 1B, for tested cell densities ranging from 10^3^ to 10^5^/ml, the growth inhibition increases with itraconazole dose, suggesting that the inhibition of tumor proliferation is dose-dependent.

The ability of tumor cells to spread is then estimated independently by counting the tumor colonies on soft agar. In Figure 1C, untreated cells form sizeable colonies and show rapid proliferation, but in the presence of 1, 2 or 4 μM itraconazole the colonies formed by A375 and SK-MEL-28 cells are fewer and smaller. For example, at doses of 2 μM and 4 μM, the number of colonies with a respective diameters of ≥ 25μm and ≥50 μm decreases by more than 50% as shown in Figure 1C. Again, our results indicate a significant inhibitory effect of itraconazole on melanoma cell proliferation.

### Itraconazole modulates gene expression of Hedgehog and Wnt pathways in melanoma cells

**Figure 2:**
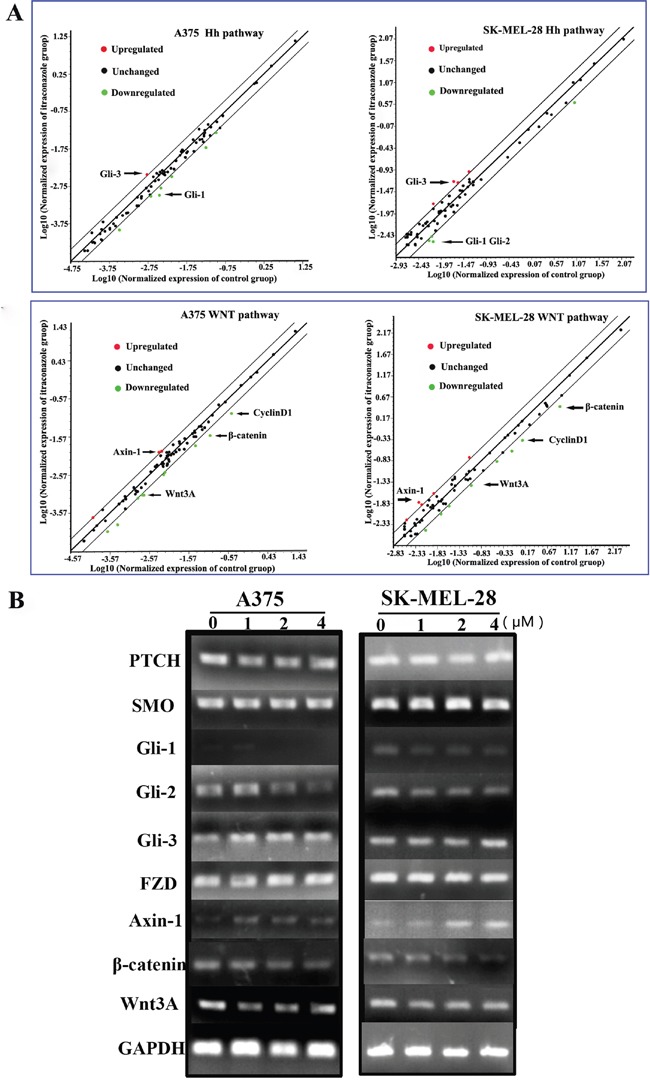
RT2 Profiler™ PCR Array screening and genes expression in melanoma cells **A**. A375 and SK-MEL-28 cells were cultured as control or under itraconazole 2 μM condition for 48 h. Total RNA was isolated from these cells and screened for mRNA expression of RT Profiler array of 84 genes using real-time PCR. The Array data were normalized with housekeeping gene panel B2M, HPRT1, RPLP0, GAPDH &β-actin and differential gene expression represented as scatter plot analysis (Red—upregulated; Black—unchanged; Green—downregulated); **B**. A375 and SK-MEL-28 cells were subjected to itraconazole for 48 h and total RNA isolated from these groups was further analyzed for specific gene mRNA expression using RT-PCR. GAPDH was used as internal control.

After A375 and SK-MEL-28 cells were treated with 2 μM itraconazole for 48 h, total RNA isolated from these cells was used to screen RT Profiler array consisting of 84 potential genes by quantitative PCR analysis in order to understand the molecular mechanisms of itraconazole on melanoma proliferation. The online analysis of gene expression is presented as scatter plots in Figure 2A. We find that itraconazole increases Gli-3, Axin-1 expression but decreases Gli-1, Gli-2, Axin-1, β-catenin, Wnt3A and Cyclin D1 expression in both melanoma cell lines when compared with untreated cells.

The RT Profiler array was then validated by RT-PCR. Similar results are found in itraconazole treated A375 and SK-MEL-28 cells. In the presence of itraconazole, the gene expression levels of Gli-3 and Axin-1 increased while Gli-1, Gli-2, β-catenin and Wnt3A transcriptionally decreased in A375 and SK-MEL-28 cells compared to untreated cells, respectively. (Figure 2B). At same time, no significant changes are seen in expression levels of PTCH, SMO and FZD in contrast to GAPDH, as calculated and shown in Supplementary Figure 1.

### Itraconazole modulates protein expression of Hedgehog and Wnt pathways in melanoma cells

**Figure 3:**
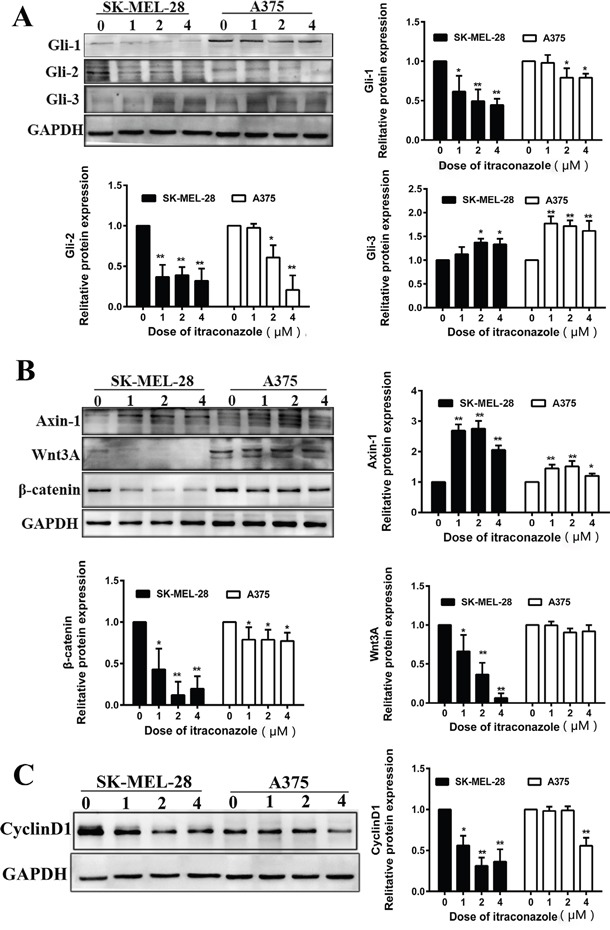
Effect of itraconazole on Wnt/β-catenin, Hedgehog signaling pathway and expression of cyclin D1 in A375 and SK-MEL-28 cells Protein was extracted from melanoma cells treated with itraconazole (0, 1, 2, and 4 μM) for 48 hr. Western blot analysis with antibodies against specific protein, including GAPDH, was performed. **A**. Western blot analysis of Gli-1, Gli-2 and Gli-3 in A375 and SK-MEL-28 cells. **B**. Western blot analysis of Axin-1, Wnt3A and β-catenin in A375 and SK-MEL-28 cells. **C**. Western blot analysis of cyclin D1 in A375 and SK-MEL-28 cells. Relative Western blot quantification of proteins normalized with GAPDH and relative to control group (value 1). Data were obtained from three independent experiments performed in duplicate and are expressed as mean ± SD (** P < 0.01 vs control, *P < 0.05 vs control).

The effects of itraconazole on Hedgehog and Wnt pathways also verified at translational level by Western blot analysis after cells were exposed to itraconazole (0, 1, 2 or 4 μM) for 48 h. In consistence with gene expression profiles, itraconazole treatment causes significant reductions of Gli-1 and Gli-2 and increase of Gli-3 protein in both A375 and SK-MEL-28 cells as shown in Figure 3A. The latter is a transcription repressor that regulates gene transcription of Hedgehog signaling pathway, the increase of Gli-3 therefore indicates that itraconazole is able to suppress Hedgehog pathway. However, the dose-dependent effect of itraconazole on this repressor is not clear, especially between 2 and 4 μM concentration of itraconazole used. Again, the inhibitory effects of itraconazole on SK-MEL-28 cells are more significant than those on A375.

**Figure 4:**
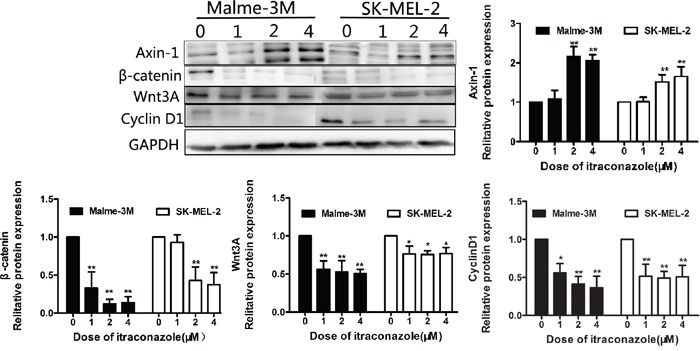
Effect of itraconazole on Wnt/β-catenin and expression of cyclin D1 in SK-MEL-2 and Malme-3M cells Protein was extracted from melanoma cells treated with itraconazole (0, 1, 2, and 4 μM) for 48 hr. Western blot analysis with antibodies against specific protein, including GAPDH, was performed. Western blot analysis of Axin-1, Wnt3A and β-catenin and cyclin D1in SK-MEL-2 and Malme-3M cells. Relative Western blot quantification of proteins normalized with GAPDH and relative to control group (value 1). Data were obtained from three independent experiments performed in duplicate and are expressed as mean ± SD (** P < 0.01 vs control, *P < 0.05 vs control).

To assess the functional relevance of Wnt/β-catenin signaling in the context of itraconazole inhibitory cell-growth, we analyzed the effects of itraconazole on the Wnt protein and its repressor Axin-1. We find that Wnt3A (Wnt growth factor protein) declines dramatically in SK-MEL-28 cells when treated with 1 and 2 μM itraconazole and is barely detectable when the dose is raised to 4 μM. Meanwhile, β-catenin is also down-regulated that coincides with an increase of one endogenous WNT inhibitor (Axin-1) in itraconazole-treated cells (Figure 3B). In consistence of a less effectiveness of itraconazole on A375 cell growth inhibition (Figure 1A), Wnt/β-catenin signaling responses are also subtle in A375 cells no matter what dose of itraconazole has been or not used as shown in Figure 3B. However, further study shows that such Wnt/β-catenin signaling responses are easily seen in SK-MEL-2 and Malme-3M cells as well (Figure 4).

### Itraconazole inhibits cyclin D1 and mTOR pathway in melanoma cells

The effect of itraconazole treatment on the expression of cyclin D1 was determined in A375 and SK-MEL-28 melanoma cell lines at 48 hours. In A375 and SK-MEL-28 cells, cyclin D1 is decreased by itraconazole treatment, indicating a cell cycle arrest. The dose-dependent behavior is also clearly reflected in SK-MEL-28 cells, which appear under all circumstances to be the cell line that is more sensitive to itraconazole (Figure 3C). Then, a reduced expression of cyclin D1 are also verified in SK-MEL-2 and Malme-3M cells (Figure 4).

**Figure 5:**
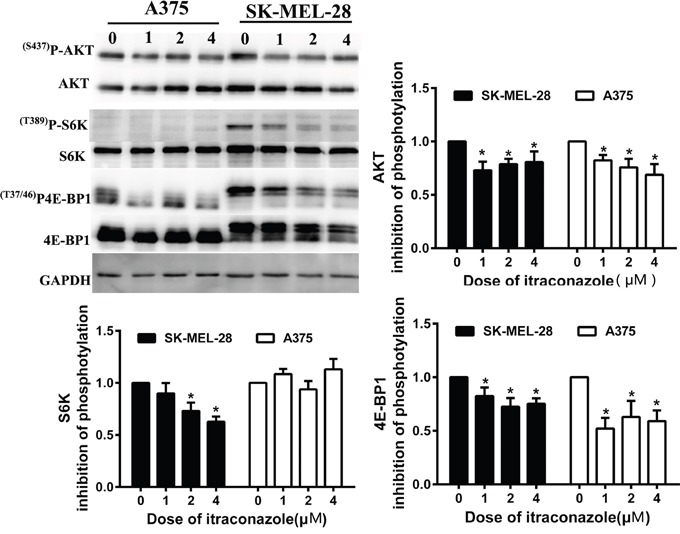
Effect of itraconazole on mTOR pathway in melanoma cells A375 and SK-MEL-28 Cells were treated with the indicated concentration of itraconazole for 48 h before lysis. Western blot analysis was performed with antibodies specific for phospho-AKT (p^(S473)^AKT), total AKT; phospho-S6K(^(T389)^pS6K), and total S6K; phospho-4E-BP1(^(T37/46)^P-4E-BP1), and total 4E-BP1. The effect of itraconazole on AKT, S6K, and 4E-BP1 phosphorylation in A375 and SK-MEL-28cells was quantitated. Total AKT, S6K, and 4E-BP1 values served to normalize p-AKT, p-S6K, and p4E-BP1 values to correct for differences in protein loading. After the initial subtraction of the background signal, the ratios of pAKT to total AKT, of pS6K to total S6K and of p4E-BP1 to 4E-BP1 were determined. The value for the control sample (DMSO) was set to 100% (or 0% inhibition), and the values for the itraconazole-treated samples were expressed as inhibition relative to the control sample. Data were obtained from three independent experiments performed in duplicate and are expressed as mean ± SD (*P < 0.05 vs control).

To further explain the cell cycle arrest, we also investigate the effect of itraconazole on the mTOR pathway in these melanoma cells by Western blot assay. We find that itraconazole, like rapamycin, is able to suppress the phosphorylation of p70S6K (S6K1) and 4E-BP1 in a dose-dependent manner (Figure 5). In contrast, itraconazole has no effect on the phosphorylation of either MEK or ERK (Supplementary Figure 2), indicating a high specificity of itraconazole on the mTOR pathway. Also, we tested the phosphorylation state of Akt that is presumably activated by mTORC2 [24, 25]. Unlike p70S6K, the phosphorylation of Akt at Ser473 is only slightly reduced after itraconazole treatment (Figure 5). These results suggest that itraconazole may inhibit both mTORC1 and mTORC2, and inhibition of mTORC2 probably occurs only as a direct consequence of mTORC1 inhibition.

### Itraconazole inhibits melanoma growth and extends survival of xenograft mice *in vivo*

For *in vivo* efficacy of itraconazole on melanoma, athymic nude mice were subcutaneously implanted with A375 melanoma cells and divided into five groups. Each group comprising 7, 7, 6, 6 or 6 mice was administered with vehicle or itraconazole by oral gavage. The first group (vehicle) received 40% (w/v) 2-hydroxypropyl-b-cyclodextrin (HPCD) solution, whereas the other four groups received itraconazole at respective doses of 100, 75, 50, and 30 mg/kg twice a day per animal. The mice tested in this experiment showed only a slight intolerance to the twice daily regimen with respect to weight loss. (Toxicity was defined as ≥20% of mice showing ≥20% body weight loss and/or mortality) (Supplementary Figures 3). At the beginning of itraconazole treatment, small tumors appeared at 7 days after tumor implantation in all the mice in the sizes of ~100 mm^3^.

**Figure 6:**
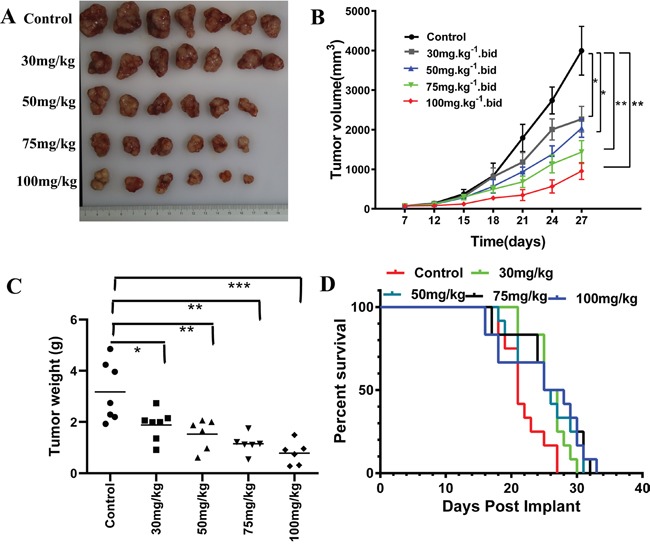
Effect of itraconazole on A375 melanoma xenograft and survival of athymic nude mice **A**. Photographs of excised tumors from each group. **B**. Average tumor volume of control and itraconazole-treated mice plotted over days after tumor cell inoculation. Mice were treated with vehicle control (40% cyclodextrin orally twice daily, n = 7 tumors; black), 30mg/kg, (itraconazole orally twice daily, n = 7; gray), 50mg/kg (itraconazole orally twice daily, n = 6 tumors; blue), 75mg/kg (itraconazole orally twice daily, n = 6 tumors; green), or 100mg/kg (itraconazole orally twice daily, n = 6 tumors; red), Data represent group means ± SD (*p < 0.01 versus control; **p < 0.01 versus control). **C**. Average weight of tumor in control and itraconazole-treated mice. Data represent group means (*p < 0.01 versus control; **p < 0.01 versus control; ***p < 0.01 versus control). **D**. Kaplan-Meier survival analysis of an melanoma xenograft with vehicle control (n = 12), itraconazole 30mg/kg (n = 12), itraconazole 50mg/kg (n = 12), itraconazole 75mg/kg(n = 12), or itraconazole 100mg/kg(n = 12).

The tumor growth in mice treated with itraconazole are generally suppressed with our tested dosage range as shown in Figures 6A & 6B, particularly in animals receiving high doses. The inhibitory effect is dose-dependent within the 20 day regimen. Compared to the average tumor volume at day 27 of 3995.0 mm^3^ in mice receiving vehicle only, the average tumor volume for mice receiving 30 mg/kg itraconazole is 2266.2 mm^3^, which is decreased by 43.27% (*P*=0.0325). The tumor volumes are further suppressed with higher dosages of itraconazole, giving the average sizes to 2040.1 mm3, 1434.6 mm3 and 955.8 mm^3^ for 50, 75 and 100 mg/kg treatments, respectively. The tumor growth is reduced by 48.9% for 50 mg/kg (*P=0.0214*), 64.1% for 75 mg/kg (*P=0.0147*) and 76.07% for 100 mg/kg itraconazole (*P=0.0036*) at day 27. At the same time, the tumor weights in itraconazole treated mice are consistently decreased in a dose-dependent manner *in vivo* as seen in Figure 6C.

The possible clinical outcomes of anti-melanoma effects of itraconazole were also estimated by survival measurement in this xenograft model. We find that the median survival times of mice receiving itraconazole (Figure 6D) are longer than the control group (21 days) as 26 days (30 mg/kg, p = 0.006), 26 days (50 mg/kg, p = 0.0364), 26.5 days (75 mg/kg, *p =0.0069*), 26.5 days (100 mg/kg, p =0.0313) (Figure 6D). Since the improvement of survival time is quite limited with the dose increasing, we recommend using a lower and effective dose of itraconazole to minimize toxicity (Supplementary Figure 3, 4).

**Figure 7:**
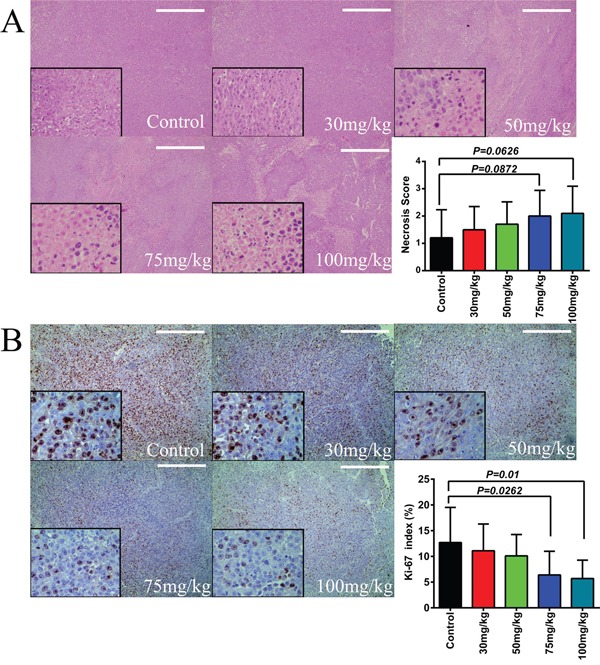
Effect of itraconazole on tissue necrosis and expression of Ki-67 in melanoma *in vivo* **A**. Itraconazole slightly increases necrosis of melanoma. From the tumor growth inhibition study, melanoma from itraconazole-treated mice show more evidence of necrosis than cyclodextrin control treated tumors as measured by necrosis score with higher numbers indicating worsening necrosis. Images are H&E sections ( ×100 and ×400). Graphical representation of necrosis score counted from five HPFs (100×) per section of six different slides from each animal group. The scale bar represents 100μm. **B**. Itraconazole reduces Ki-67 expression in melanoma *in vivo*. Representative staining of Ki-67 (×100 and ×400) Six slides from each group were stained. Graphical representation of percentage of positive cells (Ki-67 index) counted from five HPFs (400×) per section of six different slides from each animal group. The scale bar represents 100μm.

Melanoma inhibition *in vivo* is further assessed by measurement of Ki-67 levels in the tumor masses. The Ki-67 protein (also known as MKI67) is a cellular marker for cell proliferation and has been widely used in immunohistochemical assays. By counting the positive cells from five HPF (400×) fields per section of total six sections per mouse (Figure 7B), our results show that itraconazole treatment is able to inhibit Ki-67 in a dose-dependent manner (Figure 7B). These results strongly suggest that itraconazole treatment can suppress tumor cell proliferation and inhibit tumor spread *in vivo* as well. Histologically, the resulting masses in this xenograft model exhibit certain extents of necrosis in tumors excised from mice that had been treated with itraconazole (Figure 7A). However, no statistical significance can be found between itraconazole-treated and medium-treated mice under microscopy.

### Toxicity of itraconazole in mice

Although itraconazole has a strong safety record for treatment of human diseases, to answer whether itraconazole given to xenograft melanoma mice in this experiment can cause cytotoxicity, the plasma from itraconazole free- and itraconazole treated-mice were subjected to a panel of organ functional tests including hepatic alanine aminotransferase [ALT], total bilirubin [TBIL], and alkaline phosphatase [ALKP] and kidney functional tests such as contents of creatinine, urea nitrogen [BUN], albumin, cholesterol, glucose, and calcium in the blood samples.

The mice under high doses of itraconazole (75 mg/kg or 100mg/kg) experienced some liver function impairments, as average levels of ALT and ALKP were slightly higher in these mice compared to mice with lower or no doses of itraconazole even though no statistical significance (Supplementary Figure 4A & 4B) is evident. On the other hand, albumin and TBIL are similar among the groups (Supplementary Figure 4C & 4D). For kidney function, creatinine and BUN are well maintained within normal ranges (Supplementary Figure 4E & 4F). However, cholesterol levels are significantly increased in mice administered with 75 mg/kg or 100 mg/kg itraconazole, as shown in Supplementary Figure 4G, indicating that itraconazole interferes with cholesterol metabolism at these higher doses. Again, both anti-melanoma effects and side effects should be taken into account for proper dose calibration in future practice.

## DISCUSSION

Malignant melanoma is the deadliest form of skin cancer. The incidence of the disease continues to rise faster and mortality rates for melanoma remain generally higher than any other cancer type, with a 5-year survival rate of only 15% for the patients at advanced phases. The long-term prognosis is extremely poor for this disease [26].

Although much progress has been made in cancer therapies including melanoma, challenges for melanoma treatment still remain due to resistance and the low specificity of drugs used currently and the lack of new drug candidates. As soon as itraconazole had been identified as a novel inhibitor of Hh and angiogenesis [9, 10], a number of phase 2 clinical trials and retrospective analyses were initiated to investigate its anti-cancer effectiveness. The aim of this study is to investigate the possibility of using itraconazole as an anti- melanoma agent for therapeutic purposes.

The link between Hedgehog (Hh) signaling pathway and human cancers has long been recognized [27]. For example, the critical roles of Hh signaling in the development of basal cell carcinoma (BCC) have been convincingly demonstrated in genetic mutation analyses, mouse models, and clinical trials of BCCs using Hh signaling inhibitors [28]. However, the activity of the Hh pathway in melanoma tumor genesis was not revealed until recent years [29] due to the lack of genetic alterations in Hh pathway genes [30] and the lack of mouse models for Hh signaling-mediated melanoma [31]. Likely, the Hh signaling pathway contributes to melanoma progression, as suggested by the delayed tumor growth of B16F0 melanoma cells in immunodeficient mice and also by tumor size reduction in a melanoma transgenic mouse model with elevated Gli-1 that was induced by oncogenic N-RAS when treated with Hh inhibitor [6]. In consistent with the results from mouse model, the Gli-1 expression also correlates with tumor progression and metastasis in human melanomas [32].

Other downstream counterparts of Hh signaling pathway such as Gli-2 and Gli-3 have been associated with melanoma as well. The high expression of Gli-2 was accompanied by invasive and metastatic phenotypes in both *in vitro* and *in vivo* studies [33]. Gli-3 is a gene transcription repressor and its up-regulation can effectively inhibit the Hh-mediated progression of tumors [34]. Previous studies on the mechanism of itraconazole on BCC revealed a reduction of Gli-1 in itraconazole treated-BCC cells [10]. In this study with melanoma models, our data clearly show that down-regulated Gli-1 and Gli-2, and up-regulated Gli-3 in melanoma cells and mice treated with itraconazole are consistent with the reduced proliferation of melanoma cells and the smaller size of tumors in mice model, suggesting that itraconazole can serve as a potent inhibitor of Hh signaling to aid in melanoma therapy when combined with other drugs. Hh intracellular signaling pathway binds to SMO and leads to inhibition of an aberrant activation of the Hh pathway. Itraconazole acts as an inhibitor of Hh pathway that binds to SMO. Since SMO has similar structure with FZD, an upstream modulator of Wnt/β-catenin signaling pathway, which raises a question whether the FZD-Wnt pathway is also involved in the anti-melanoma effects of itraconazole [35]. In the present study we find that itraconazole can decrease Wnt3A and β-catenin by changing transcription and protein levels in A375, SK-MEL-28, SK-MEL-2 and Malme-3M melanoma cells. In other tumor types such as colorectal and breast cancer, activation of the Wnt/β-catenin pathway is often accompanied by increased tumor genesis, tumor cell proliferation and decreased patient survival [36–38], and activation of this pathway can be interrupted by chemotherapy-induced apoptosis [39–41]. While the Wnt/β-catenin pathway was down-regulated by small interfering RNAs (siRNA), apoptosis can be induced in a variety of human cancer cells, indicating that the Wnt/β-catenin pathway may be associated with the apoptotic process. This correlation was also observed in the pathogenesis of malignant melanoma [42, 43]. In about one-third of melanoma, aberrant activation of the Wnt/β-catenin pathway leads to a very poor prognosis [44]. Melanoma metastasis is often associated with activation of the Wnt/β-catenin signaling pathway [45]. Despite these promising characteristics, indications are sometimes contradictory concerning the role of the Wnt/β-catenin pathway in melanoma development. For example, one study has shown that activation of the Wnt pathway in melanoma may be of therapeutic benefit [38]. In addition, Axin-1, a negative regulator of β-catenin, was also linked to tumor progression, including melanoma [46]. We find that the expression of Axin-1 is up-regulated in this study. Consistent with the suppression of β-catenin and Wnt3A, we conclude that anti-proliferative effects of itraconazole on tumor cells are facilitated by modulating Wnt3A and β-catenin, and negatively regulate gene Axin-1.

The phosphoinositide 3-kinase (PI3K)-AKT-mTOR signaling pathway plays an important role not only in the regulation of some crucial physiological cell processes – including cell-cycle progression, differentiation, ribosomal biogenesis and protein translation – but also in the regulation of certain aspects of tumor behavior such as cell growth, survival and chemoresistance [47–49]. This pathway has been closely associated with the initiation and progression of many tumors, including melanoma [47–49]. An imbalance in the PI3K-AKT pathway can cause aberrant activation of the mammalian target of rapamycin (mTOR), one of the most-extensively studied downstream effectors of this pathway [50]. In mammalian cells, mTOR is found in two functionally distinct protein complexes – mTOR complex 1 (mTORC1) and mTORC2 [51]. The protein complex (mTORC1), consisting of mTOR, raptor and mLST8 serving as the upstream kinase of p70S6K and 4E-BP1, regulates cellular growth by integrating signals from growth factor receptors and intracellular nutrients [52–55]. The second protein complex mTORC2, composed of mTOR, Rictor, Sin1 and others serves as the upstream kinase of AKT that is pivotal in regulating cellular growth, migration and survival [56]. When mTORC1 activity is blocked by rapamycin and its analogs, an encouraging clinical efficacy has been seen in some types of cancers [50, 57], but the effects are somewhat more modest in other cancers including melanoma in pre-clinical settings and clinical trials [48, 58]. This may be largely due to the feedback activation of the PI3K, mTORC2 and Erk-MAPK signaling pathways upon mTORC1 inhibition [48, 57]. It has been reported that itraconazole specifically inhibits the mTOR pathway in endothelial cells via an antiangiogenic mechanism [59]. We find that itraconazole can inhibit the activation of mTORC1 effectively in melanoma cells, indicated by decreased p-S6K and p-4E-BP1 levels in Western blots, and attenuate mTORC2 activity as well, indicated by less p-AKT-Ser473 under itraconazole treatment. These results suggest that itraconazole may act as a dual inhibitor for the PI3K-mTOR pathway in melanoma cells.

In mice xenograft model, we demonstrate that this compound can inhibit tumor cell proliferation and repress tumor colony formation *in vitro*, which is consistent with reduction of tumor size and prolonged survival in melanoma xenograft mice. Study of the mechanism of anti-melanoma effects reveals that itraconazole acts on the suppression of Hh, Wnt and AKT-mTOR pathways. Thus, rational combination of this compound with other chemo drugs that may target some specific tumorigenic mechanism will be an effective strategy to enhance conventional cancer therapy. The response doses of itraconazole in mice examined in this experiment are indeed higher than the commonly used therapeutic doses (i.e., 400 mg/day orally for 15 days) in patients with fungal infections, but are similar to itraconazole doses in patients with severe fungal infections [60, 61], In these cases, a high dose of itraconazole ranging from 600 to 900 mg/day can be given to patients for 3 to 16 months with close monitoring for any toxicity of this compound. With high dose itraconazole in our mice model (75 and 100 mg/kg), we find no signs of severe liver and kidney damage except a slight elevation of serum cholesterol during the 20 days of treatment. In fact, the toxicity data suggests that the side reactions of this compound are reversible with dose adjustment [62].

Taken together, our study is the first to evaluate itraconazole as possible inhibitor for melanoma in both *in vitro* and *in vivo* models. The results suggest that this agent has a potent anti-melanoma feature and may be very useful to synergize other anti-cancer drugs by blocking the Hh, Wnt and PI3K/mTOR signaling pathway in melanoma cells. Strategically, this may enhance the therapeutic efficacy and prevent development of chemo resistance.

## MATERIALS AND METHODS

### Cell culture and reagents

A375 and SK-MEL-28 melanoma cell lines were obtained from American Type Culture Collection (ATCC) and were cultured in DMEM or RPMI 1640 medium (Kengene, Nanjing, China) respectively. All culture media contain 10% FBS, 100 U/mL streptomycin and penicillin. Cells were cultured at 37°C in a humidified atmosphere with 5% CO_2_. Itraconazole (Sigma-Aldrich) was dissolved in dimethylsulfoxide (DMSO) for all *in vitro* experiments. Itraconazole oral solution (10 mg/mL, Sporanox, Ortho Biotech) was obtained from the Skin Disease Hospital of Chinese Academy of Medical Sciences for *in vivo* experiments.

### Cell proliferation assay

CCK-8 assay: A375 and SK-MEL-28 melanoma cells were seeded at a density of 6×10^3^cells/well in a 96-well plate and were treated with itraconazole (～64 μM) in FBS-free medium, for 24, 48, and 72 hours. Then cells were incubated with the Cell Counting Kit-8 solution (Yeasen, Shanghai, China) for two hours. Light absorbance at a wavelength of 450nm was measured using a Spectrra MAX 190 absorbance microplate reader (Molecular Devices, California, USA). Experiments were repeated at least three times in duplicates.

Cell counting assay: A375 and SK-MEL-28 melanoma cells were seeded at indicated density(1×10^3^, 1×10^4^, 1×10^5^ cells/well)in a 6-well plate and were treated with itraconazole (0, 1, 2, 4μM) in FBS-free medium, for 48 hours. Then, cells were continuously cultured in medium containing 10% FBS without itraconazole for additional 5 days (1×10^4^, 1×10^5^ cells/wel) or 8 days (1×10^3^). Cells were stained with crystal violet for 30 min and pictures were taken in at least five random microscopic fields. Then, numbers of cells were counted by Adobe Photoshop CS5 software (Adobe, USA). Experiments were repeated three times in duplicates.

### Colony formation assay in soft agar

A375 and SK-MEL-28 melanoma cells in logarithmic growth phase were trypsinized and suspended into a single cell suspension. Bottom layer agar (0.6%) preparation: 1.5 mL 1.2% agar was added into 1.5 mL 2X culture medium (with 20% lipid-depleted serum, 2X antibiotics, 2X itraconazole 2, 4, 8 μM), mixed well, laid onto a 6-well plate and allow to solidify at room temperature. Upper layer agar (0.4%) preparation: 0.5 mL of 0.8% agar was mixed, added to 2X culture medium (with 20% lipid-depleted serum, 2X antibiotics, 2X itraconazole 2, 4, 8 μM) containing 10^3^ cells, and laid onto the bottom layer. Then plates were incubated for 14 days at 37 °C with 5 % CO2. Photograph of the well plates were taken under a microscope and numbers of the colonies were calculated.

### RT^2^ Profiler™ PCR array screening

Total RNA was isolated from A375 and SK-MEL-28 melanoma cells pretreated with 2 μM itraconazole or no-treated as control, using RNeasy Mini Kit (Qiagene, Maryland, USA). The reverse transcription reaction was performed using RT2 First Strand Kit (Qiagene, Maryland, USA) in a 25 μL of reaction volume containing total RNA (2 μg), 1 × PCR buffer and 2 mM MgCl_2_, at 42 °C for 15 min followed by 95°C for 5 min. The RT^2^ Profiler PCR Array (Qiagene, Maryland, USA) that includes 84 genes was performed by qPCR using RT^2^SYBR@Green/ROX™PCR master mix in Stratagene MX3000p QPCR System (Agilent Technologies Inc., USA). Thermal cycling parameters are 95 °C for 10 min, followed by 40 cycles of amplifications at 95 °C for 15s, 60 °C for 30 s, and 72°C for 5 min as the final elongation step. Relative levels of mRNA expression were normalized in all the samples with expression levels of housekeeping genes (B2M, HPRT1, RPLP0, GAPDH and β-actin) mRNA amplification. The data are analyzed using the Data Analysis Center in a web resource (http://pcrdataanalysis.sabiosciences.com/pcr/arrayanalysis.php).

### RNA preparation and semi-quantitative PCR

Total RNA was extracted from A375 and SK-MEL-28 melanoma cells at forty-eight hours post-treatment using TRIzol reagent (Invitrogen, USA) according to the suggested instructions. cDNA was synthesized using HiScript Q RT SuperMix for qPCR(Vazyme, Nanjing, China) according to the manufacturer's instructions. RNA (2 μg) was converted to complementary DNA by One taq®Hot Start(BioLabs, England) according to the manufacturer's protocol. The PCR amplification protocol was 95°C for 5 min, followed by 30 or 35 cycles of 95°C for 20 s, 55°C for 20 s, 72°C for 30s and 72°C for 5 min as the final elongation step. Amplified products were resolved by 1% agarose gel electrophoresis, stained with ethidium bromide and photographed under ultraviolet light. Primer sequences are shown in the supplement materials. Details about the primer sequences was shown in Table 1.

**Table 1:** Primer sequences for RT-PCR are the following (5′ to 3′)

PTCH1-F, TGGGTGGAAGTTGGAGGACGAG
PTCH1-R, CCCACAATCAACTCCTCCTGCC
Smo-F, ATGGATGGTGCCCGCCGAGAG
Smo-R, ATGGTCTCGTTGATCTTGCTGG
Gli-1-F, CCCAACTCCACAGGCATAC
Gli-1-R, ACACGAACTCCTTCCGCTCC
Gli-2-F, CCCACTCCAACGAGAAACCC
Gli-2-R, TCTTTGAGCAGCGGTGTGCG
Gli-3-F, CGAACAGATGTGAGCGAGAA
Gli-3-R, TTGATCAATGAGGCCCTCTC
FZD1-F, TTCAGCAGCACATTCTGAGG
FZD1-R, CCTGCACACATTTTCCCTTT
Wnt3A-F, GGTCTCATTTGGGGGCGTTC
Wnt3A-R, TTGGCTCCAGGAAAGCGGAC
β-catenin-F, CCGACACCAAGAAGCAGAGATG
β-catenin-R, GGGACAAAGGGCAAGATTTCG
Axin1-F, ACGGTACAACGAAGCAGAGAGCT
Axin1-R, CGGATCTCCTTTGGCATTCGGTAA
GAPDH-F, AGGTGAAGGTCGGAGTCAACG
GAPDH-R, AGGGGTCATTGATGGCAACA

### Western blotting

A375 and SK-MEL-28 melanoma cells were seeded on 6-well plates in their respective medium and were treated with itraconazole (0, 1, 2, 4 μM). After treatment, medium was removed from attached cells and washed twice with phosphate-buffered saline and then lysed in RIPA lysis buffer kit (BestBio, shanghai, China), on ice for 30 min. The lysates were collected and centrifuged at 12,000 g for 20 min at 4°C. Protein concentrations were detected using a bicinchoninic acid protein assay kit (Multisciences, Hangzhou, China). Aliquots of the lysates were boiled for 5 min, electrophoresed on 10% SDS-PAGE gels and transferred to a PVDF membrane (Merck Millipore, Germany). The membrane was blocked with 1% BSA at room temperature for 1 h and then probed with primary monoclonal rabbit antibodies. Antibodies against Gli-1, Gli-2, Gli-3, Axin-1, (1:1000; Rockland, USA), Non-phospho-β-catenin (Ser33/37/Thr41), Wnt3A, P44/42MAPK(Erk1/2), phosphor-P44/42MAPK(Erk1/2), MEK1/2(47E6), phosphor-MEK1/2(Ser221), Cyclin D1(92G2) (1:1000; Cell Signaling, Beverly, MA), GAPDH (1:1000; Bioss, Beijing, China). Secondary anti-rabbit IgG was from Cell Signaling and visualized by a chemiluminescence (ECL) detection system. Densitometric analysis of developed blots was performed with the Adobe Photoshop CS5 software and expressed as a ratio of the quantity of indicated protein to β-actin, followed by standardization, with the ratio of the normal control set as 1.

### Animal

Athymic nude female mice, ages 7 to 8 weeks, weighing approximately 20 to 22 g, were purchased from the Model Animal Research Center of Nanjing University. The health of all animals was monitored daily by gross observation and analysis of blood samples of sentinel animals. All animal experiments were performed in accordance with protocols approved by the Institutional Animal Care and Use Committees and were performed in accordance with the Animals in Research: Reporting *In Vivo* Experiments (ARRIVE) guidelines for the care and usage.

### Melanoma xenografts

For the A375 xenografts, 2.5 × 10^6^ cells in 0.2 mL of PBS were injected subcutaneously (s.c.) into the right lateral flank of mice, using a 12-gauge trocar needle. The tumor cells grow and form tumors after implantation. By day seven, the average volume of tumors attained to nearly 100 mm^3^ without itraconazole treatment. Drinking water was acidified. In itraconazole group, mice with tumors were treated twice per day in 8-12 hr apart with itraconazole oral solution (10 mg/ml) containing 40% (w/v) HPCD (2-hydroxypropyl-b-cyclodextrin), at the doses of 100, 75, 50 and 30 mg/kg. Meanwhile, equal volumes of 40% (w/v) HPCD solution was gavage twice daily in control mice group. The tumor growth curve is determined by measuring the tumor volume using the equation: Volume = (L×W^2^)/2. At the end of the experiment, mice were euthanized and tumors were excised for immunohistochemistry. All the animal experiments were performed in accordance with the Animal Research: Reporting *In Vivo* Experiments (ARRIVE) guidelines for the care and usage.

### Efficacy and safety end points

Survival was evaluated using a predefined cutoff volume of 2,000 mm^3^as a surrogate for mortality. The percent increase in life span was calculated as follows: 100×[(MDD-treated tumor-bearing animals) − (MDD control tumor-bearing animals)]/MDD control tumor-bearing animals. MDD represents median day of death. Average percentage weight change was used as a surrogate end point for tolerability in all experiments. Toxicity was defined as ≥20% of mice showing ≥20% body weight loss and/or mortality [22]. The health status of animals was checked daily, and weights were recorded two to three times a week.

### Immunohistochemistry

Formalin-fixed paraffin-embedded tissue sections were prepared from xenografted tumors. Tissue samples were sliced from paraffin blocks (5-μm sections), deparaffinized three times in xylene for 5 min and hydrated in a gradient ethanol (100%, 95%, 70%, and 50%). The sections were stained with hematoxylin and eosin (H & E) for pathological examination. For immunohistochemical staining, blocking of unspecific peroxidase activity was performed for 30 min with 3% H_2_O_2_ and 90% methanol. Citrate buffer (10 mM [pH 6]) was used for antigen retrieval. The rabbit monoclonal anti-body Ki-67 (D2H10) (dilution 1:150; Cell Signaling, Beverly, MA) was used. The primary antibody was incubated overnight at 4°C. Subsequently, after being washed with PBS, the biotinylated secondary antibody was incubated for 30 min. The tissues were incubated for 1 hr in an avidin–peroxidase complex (ABC, Vector Laboratories, Inc., Burlingame, USA). DAB with 5% H_2_O_2_ was used for detection. The slides were counter-stained with hematoxylin. All Slides were examined and photographed under a light microscope.

### Necrosis scoring of melanoma

Hematoxylin and eosin stained (H&E) sections of paraffin-embedded melanoma after the growth inhibition study were examined for necrosis. Necrosis was scored as follows: 0 = no evidence of necrosis with melanoma cells; 1 = mild necrosis with nuclear pyknosis; 2 = moderate necrosis with < 25% necrosis; 3 = severe necrosis with > 25%necrosis. After evaluation of necrosis scores, pairwise comparison between control and itraconazole treatment melanoma were made using a two-sided unpaired t test with GraphPad Prism software.

### Ki-67 index

The Ki-67 index was determined in the most proliferative area. In this “hot spot”, the percentage of nuclear DAB stained cells was estimated in about 200 tumour cells. At least 10 high-power fields was selected and calculated, the average positive cells (%) was treated as Ki-67 proliferation index [23].

### Drug toxicity evaluation

Just before mice were killed, blood was collected, stayed static for at least 30 minutes and centrifuged at 3000r/min for 10 minutes to separate serum. Serum isolated from blood of mice from each group was subjected to analysis of a panel of hepatic function tests (alanine aminotransferase [ALT], total bilirubin [TBIL], alkaline phosphatase [ALKP], albumin), kidney function tests (creatinine, blood urea nitrogen [BUN], albumin), and cholesterol. All assays were performed using kits from Bioassay Systems (Hayward, CA). All assays were performed according to the manufacturer’ s instructions.

### Statistical analysis

The data are analyzed using the GraphPad Prism ver. 6.04 software (Graph-Pad Software, La Jolla, CA). Data are presented as mean ± SD. The differences are assessed by Student's t-test or one-way analysis of variance (ANOVA) in which a *p* value ≤ 0.05 is considered to be statistically significant in the ANOVA analysis. Dunnett's test is also used to compare the differences between the groups. Survival plot is generated by the Kaplan-Meier method. Survival rates of treated groups with different dosages and vehicle group are compared and evaluated by log-rank test with *p*≤ 0.05 considered as of statistically significant.

## SUPPLEMENTARY MATERIALS FIGURES AND TABLES


