# Supplementary material for: Itraconazole exerts its anti-melanoma effect by suppressing Hedgehog, Wnt, and PI3K/mTOR signaling pathways
Source: Oncotarget. 2017 Feb 14;8(17):28510–25. doi: 10.18632/oncotarget.15324 (PMC5438669; doi:10.18632/oncotarget.15324)
Supplement: Supplementary file 1 [file oncotarget-08-28510-s001.pdf]

Itraconazole exerts its anti-melanoma effect by suppressing Hedgehog, Wnt, and PI3K/mTOR signaling pathways

SUPPLEMENTARY FIGURES

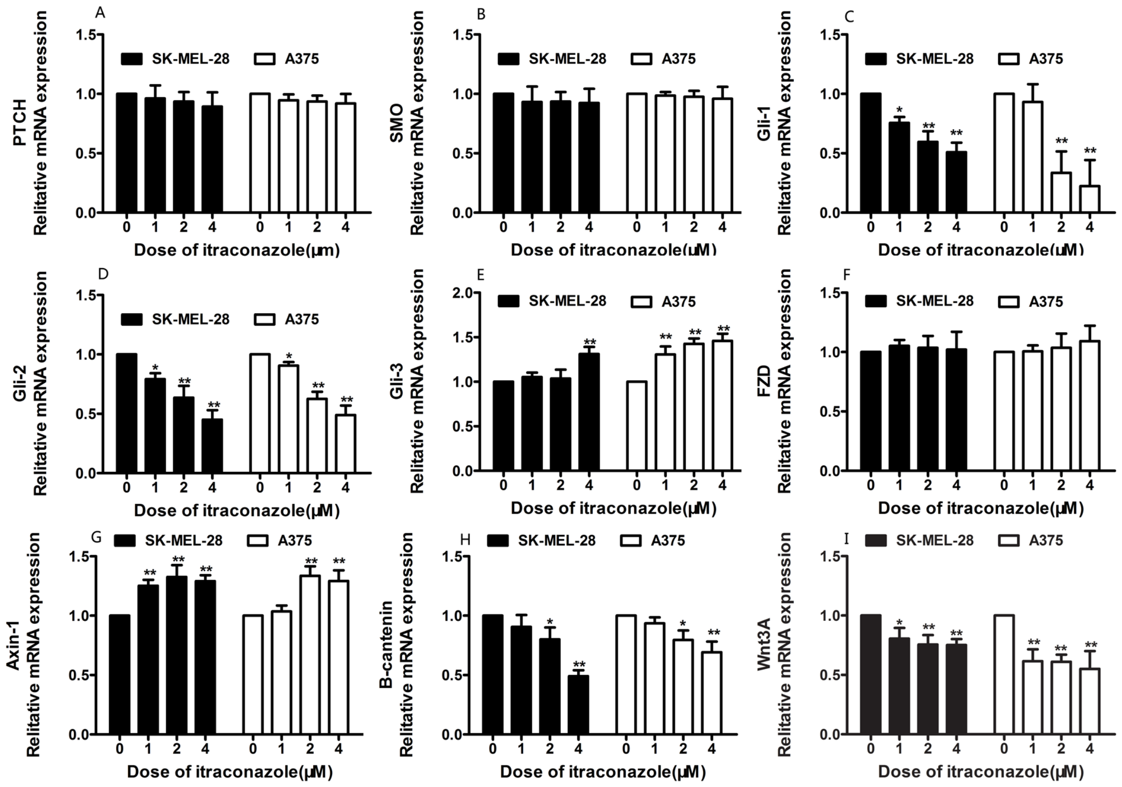

Supplementary Figure 1: The quantitative data of genes expression in melanoma cells by RT-PCR. A375 and SK-MEL-28 cells were subjected to itraconazole for 48 h and total RNA isolated from these groups was further analyzed for specific gene mRNA expression using RT-PCR. **A.** PTCH, **B.** SMO, **C.** Gli-1, **D.** Gli-2, **E.** Gli-3, **F.** FZD, **G.** Axin-1, **H.** B-catenin, **I.** Wnt3A GAPDH was used as internal control. Data were obtained from three independent experiments performed in duplicate and are expressed as mean ± SD (*P < 0.05 vs control and **P < 0.01 vs control).

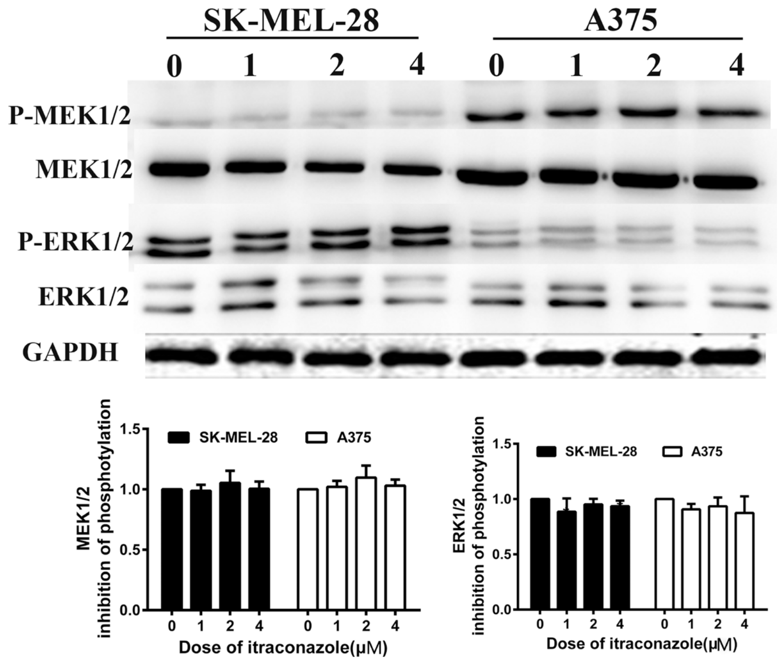

Supplementary Figure 2: Effect of itraconazole on MEK and ERK phosphorylation in melanoma cells. A375 and SK-MEL-28 Cells were treated with the indicated concentration of itraconazole for 48 h before lysis. Western blot analysis was performed with antibodies specific for phospho-MEK1/2 (pMEK1/2), total MEK1/2, phospho-ERK1/2 (pERK1/2), and total ERK1/2. The effect of itraconazole on MEK and ERK phosphorylation in A375 and SK-MEL-28cells was quantitated. Total MEK1/2 and ERK1/2 values served to normalize pMEK1/2 and pERK1/2 values to correct for differences in protein loading. After the initial subtraction of the background signal, the ratios of pMEK1/2 to total MEK1/2 and of pERK1/2 to total ERK1/2 were determined. The value for the control sample (DMSO) was set to 100% (or 0% inhibition), and the values for the itraconazole-treated samples were expressed as inhibition relative to the control sample. Data were obtained from three independent experiments performed in duplicate and are expressed as mean ± SD (*P < 0.05 vs control).

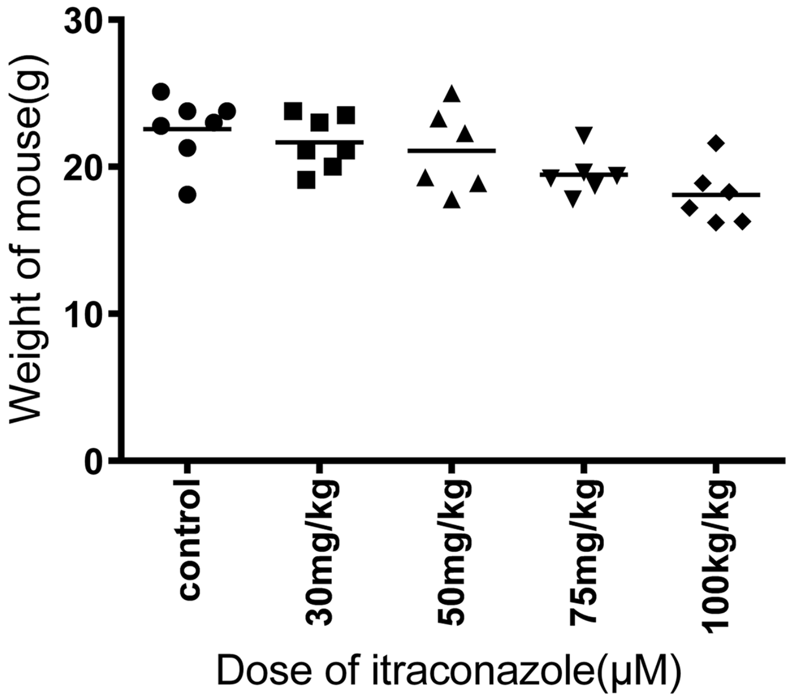

Supplementary Figure 3: Effect of itraconazole on mouse weight. Average weight of mouse in control and itraconazole-treated mice. Data represent group means

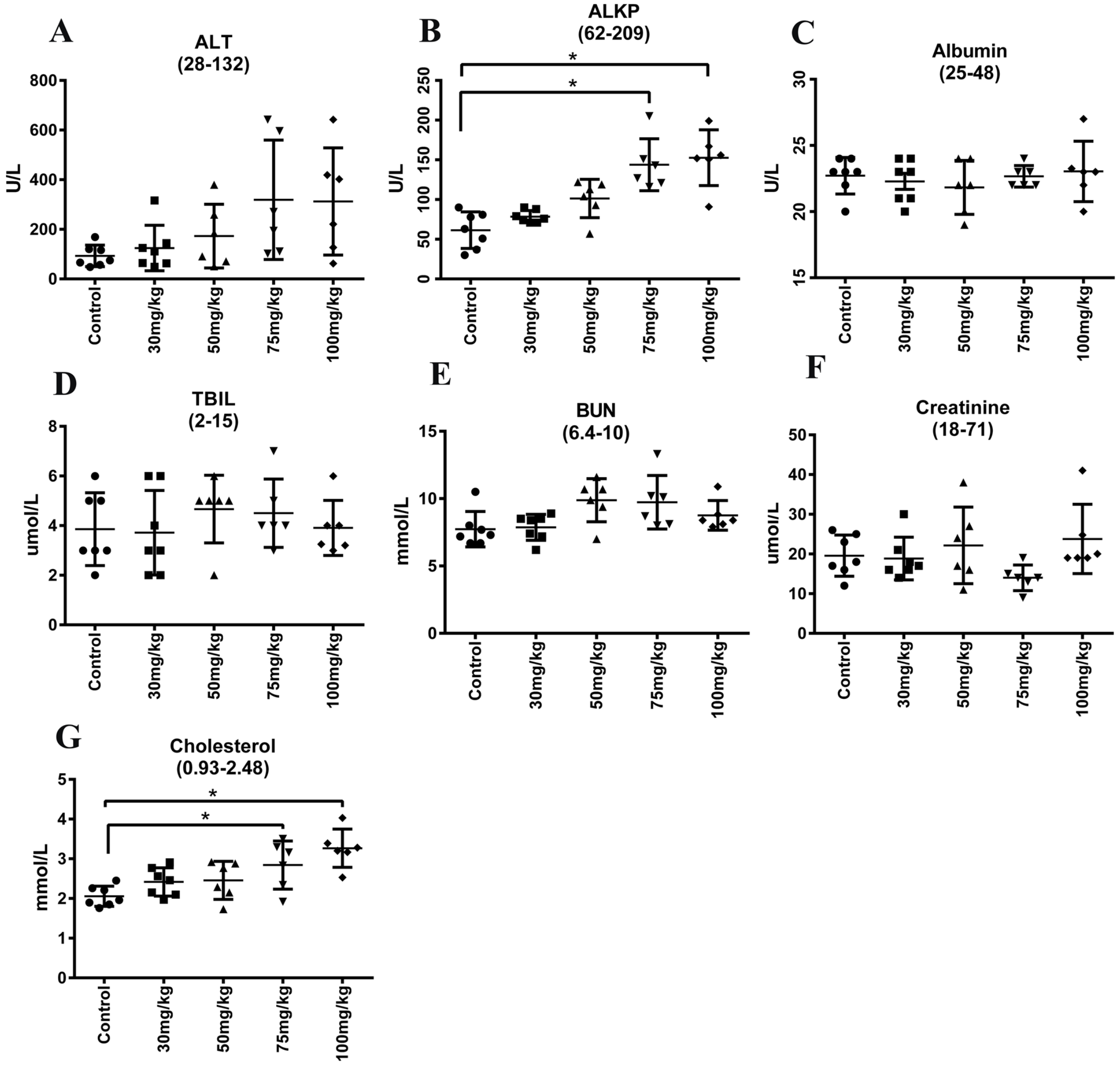

Supplementary Figure 4: Cytotoxic profile in plasma from A375 tumor-bearing mice with or without itraconazole treatment. Before sacrificing the mice at 4 weeks, blood was collected, and plasma was separated. Mouse plasma from untreated group and itraconazole 100 mg/kg, 75 mg/kg, 50 mg/kg, 30 mg/kg (M) were subjected to a panel of organ functional tests according to the manufacturer's instructions: **A.** ALT, **B.** AST, **C.** albumin, **D.** BUN (serum urea nitrogen), **E.** creatinine, **F.** glucose and **G.** cholesterol.
